# Generalized Eruptive Keratoacanthoma (GEKA) after Pfizer mRNABNT162b2 (Comirnaty^®^) COVID-19 Vaccination Successfully Treated with Cemiplimab

**DOI:** 10.3390/v16081260

**Published:** 2024-08-06

**Authors:** Proietti Ilaria, Skroza Nevena, Tolino Ersilia, Trovato Federica, Forte Felice, Dybala Agnieszka Ewa, Fiorentino Francesco, Potenza Concetta

**Affiliations:** 1 Dermatology Unit “Daniele Innocenzi”, “A. Fiorini” Hospital, via Firenze, 1, 04019 Terracina, Italy; nevena.skroza@uniroma1.it (S.N.); ersilia.tolino@uniroma1.it (T.E.); federica.trovato@uniroma1.it (T.F.); felice.forte@uiroma1.it (F.F.); agnieszka.dybala@uniroma1.it (D.A.E.); concetta.potenza@uniroma1.it (P.C.); 2 Pathology Unit, Santa Maria Goretti Hospital of Latina-ASL Latina, 04100 Latina, Italy; francesco.fiorentino@uniroma1.it

**Keywords:** keratoacanthoma, generalized eruptive keratoacanthoma, COVID-19, vaccination, cemiplimab

## Abstract

In December 2020, a major vaccination program against COVID-19 commenced in Europe with vaccines such as Pfizer’s mRNABNT162b2 (Comirnaty^®^). Subsequent reports of immediate and delayed skin reactions emerged. This study presents a case of a 64-year-old male who developed multiple keratoacanthomas approximately two weeks after receiving a second booster dose of the Pfizer vaccine. The patient, who had significant medical history of hypertension and diabetes, presented with erythematous, crateriform lesions on his limbs. A physical examination and histopathological analysis confirmed the diagnosis of Generalized Eruptive Keratoacanthoma (GEKA). Treatment involved cemiplimab I.v. 350 mg administered every three weeks. Within two months, the patient showed significant improvement, with the disappearance of all lesions. Dermoscopy and histopathological exams supported the GEKA diagnosis, which is a rare variant of multiple keratoacanthomas. This case suggests a potential immune-mediated mechanism triggered by the COVID-19 vaccine, leading to the rapid development of keratoacanthomas. Treatment with cemiplimab showed promise, highlighting the potential of immune checkpoint inhibitors in managing multiple keratoacanthomas. Further research is needed to explore the efficacy and safety of such treatments.

## 1. Introduction

In December 2020, Europe initiated a comprehensive vaccination program against COVID-19, introducing vaccines such as Pfizer’s mRNABNT162b2 (Comirnaty^®^), Moderna’s mRNA-1273 (Spikevax^®^), and AstraZeneca’s AZD1222 (Vaxzevria^®^) [1]. As the vaccination campaign progressed, reports of immediate and delayed skin reactions began to surface [2,3]. This paper details the case of a 64-year-old male who developed multiple keratoacanthomas following the administration of the Pfizer mRNABNT162b2 (Comirnaty^®^) vaccine, approximately two weeks after receiving the second booster dose. The patient was successfully treated with anti-PD1 immunotherapy (cemiplimab).

## 2. Case Presentation

In December 2022, a 64-year-old male presented to our clinic with complaints of several itchy lesions on his upper and lower limbs, which had appeared 10 days post-administration of the Pfizer mRNABNT162b2 (Comirnaty^®^) vaccine. The patient reported no additional symptoms and had no previous dermatological issues. His medical history included systemic arterial hypertension and diabetes mellitus, both well controlled with Hemoglobin A1c and fasting blood glucose levels within normal ranges. Other immunosuppressive comorbidities were excluded. The patient, an active outdoorsman, had a 30 pack-year smoking history and was not immunocompromised. His home medication regimen included metformin 1000 mg and acepril 10 mg.

**Figure 1:**
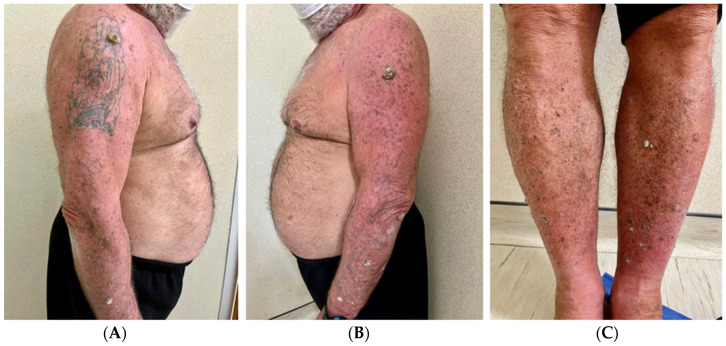
Clinical images taken during the first visit (December 2022). It is possible to note the multiple keratoacanthomas on the arms (**A**,**B**), including the vaccine injection site (**B**), and the legs (**C**), bilaterally.

**Figure 2:**
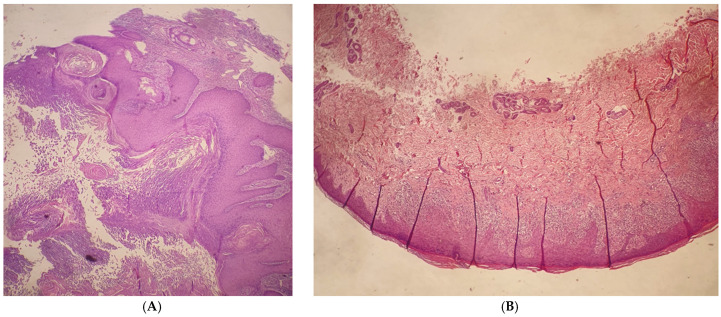
(**A**): H&E Stain, 5× magnification. A large, well-differentiated squamous tumor with a central keratin-filled crater and surrounding epidermis forms a lip around the invaginating crateriform tumor. (**B**): H&E Stain, 25× magnification. Epidermis with parakeratotic hyperkeratosis. There is a dense, band-like lymphocytic infiltrate in the dermis that obscures the dermo-epidermal junction, cytoplasmic vacuolization of basal keratinocytes, and apoptotic keratinocytes that degenerate into colloid bodies.

**Figure 3:**
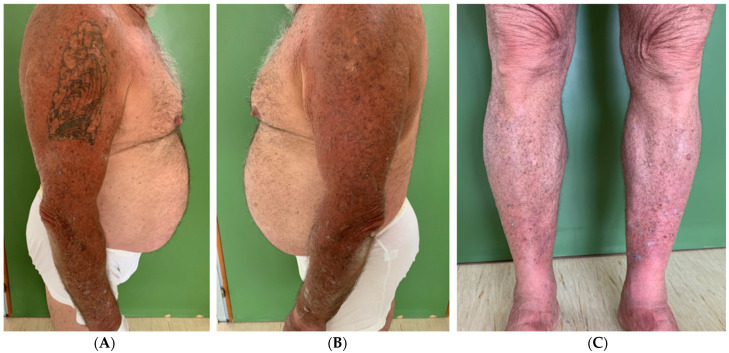
Clinical images taken at the end of treatment (March 2023). It is possible to note the regression of the multiple keratoacanthomas on the arms (**A**,**B**) and legs (**C**), bilaterally.

A physical examination revealed dome-shaped, erythematous, crateriform lesions, 1–2 cm in diameter, on both the upper and lower limbs. There were approximately six lesions on the left arm, four on the right arm, seven on the left leg, and four on the right leg, with additional lesions at the vaccine injection site (Figure 1A–C). Dermoscopy indicated central areas of superficial scales and brownish-yellow crusts, surrounded by whitish, structureless zones against a background of chronically sun-damaged skin. Histopathological examination of an excised lesion showed a circumscribed proliferation of well-differentiated keratinocytes, consistent with keratoacanthoma (KA) (Figure 2A,B). Given the clinical presentation, the diagnosis of Generalized Eruptive Keratoacanthoma (GEKA) of Grzybowski was made, and treatment with cemiplimab 350 mg intravenously every three weeks was initiated (prior authorization for off-label treatment was secured from an ethics committee.). Significant improvement was observed within two months, with complete resolution of all lesions (Figure 3A–C).

## 3. Discussion

Generalized Eruptive Keratoacanthoma (GEKA) is a rare variant of multiple KA affecting the skin and mucous membranes. Although a few cases of GEKA have been documented, there is one report of multiple keratoacanthomas arising post-vaccination with an anti-COVID-19 mRNA vaccine [4]. The etiology of GEKA remains unclear; however, excessive UV radiation, chemical carcinogens, viruses, trauma, and immunologic abnormalities have been implicated in its pathogenesis [4]. Multiple KA can also occur in genetic syndromes such as Muir–Torre syndrome, Ferguson-Smith disease, Grzybowski syndrome, incontinentia pigmenti, and xeroderma pigmentosum [5,6,7].

Immunological alterations may play a role in the development of GEKA. Several studies have investigated the relationship between GEKA onset and HPV (Human Papilloma Virus), revealing that the development of keratoacanthoma at various stages is influenced by the ratio of T helper 17 cells to T regulatory cells and the balance between pro-inflammatory and anti-inflammatory responses [5,6,7,8]. It is plausible that the rapid development of multiple keratoacanthomas in our patient was triggered by an immune-mediated mechanism induced by vaccination, which exerts a pro-inflammatory effect. The literature indicates that anti-COVID-19 mRNA vaccines can elicit robust T- and B-cell responses against SARS-CoV-2, leading to significant immune system activation [9,10,11].

Various treatment modalities for GEKA, including keratinolytic, ablative, immunomodulatory, and antiproliferative approaches, have yielded inconsistent results, highlighting the therapeutic challenges associated with extensive or multiple lesions [7]. Topical treatments are generally impractical due to the widespread nature of the disease. Surgical excision, cryotherapy, electrodessication, radiotherapy, and laser ablation are also often unsuitable, particularly in elderly patients with comorbidities and associated surgical risks. Systemic therapies, such as disease-modifying antirheumatic drugs (DMARDs) like acitretin, methotrexate, cyclophosphamide, steroids, 5-fluorouracil, and aminopterin, have shown variable efficacy, frequent adverse effects, and high recurrence rates [8,9,10].

Given the histopathological similarities between KA and squamous cell carcinoma (SCC), therapeutic strategies involving epidermal growth factor receptor (EGFR) inhibitors (e.g., cetuximab) or immune checkpoint inhibitors (e.g., cemiplimab) appear rational [11,12,13]. Although the efficacy of cemiplimab in treating multiple KA has not been demonstrated in vivo, immune checkpoint inhibitors targeting PD-1 can block PD-1/PD-L1 interactions, thereby enhancing anti-tumor T-cell responses. These inhibitors have proven effective in various neoplasms, including cutaneous SCC (cSCC), malignant melanoma (MM), non-small cell lung carcinoma (NSCLC), and Merkel cell carcinoma (MCC) [13]. Studies have shown that cemiplimab 350 mg every three weeks yields an objective response rate of 50%, a complete response rate of 8%, and a partial response rate of 43% in cSCC patients [14].

The limited literature and rarity of similar cases make it difficult to establish a definitive mechanism or strength of association between eruptive keratoacanthoma and the COVID-19 vaccine. However, given the documented cutaneous eruptions post-vaccination and the temporal association in our case, it is plausible that the vaccine may have triggered an immunological imbalance in a predisposed patient with factors such as photodamaged skin and diabetes. Further research is needed to elucidate the underlying mechanisms and establish clearer connections. 

## Data Availability

The authors confirm that the data supporting the findings of this study are available within the article.
